# Discovering the molecular differences between right- and left-sided colon cancer using machine learning methods

**DOI:** 10.1186/s12885-020-07507-8

**Published:** 2020-10-19

**Authors:** Yimei Jiang, Xiaowei Yan, Kun Liu, Yiqing Shi, Changgang Wang, Jiele Hu, You Li, Qinghua Wu, Ming Xiang, Ren Zhao

**Affiliations:** Department of General Surgery, Ruijin Hospital North, Shanghai Jiaotong University School of Medicine, Shanghai, 201801 China

**Keywords:** Left-sided colon cancer, Right-sided colon cancer, Machine learning, Mutations, Gene expression

## Abstract

**Background:**

In recent years, the differences between left-sided colon cancer (LCC) and right-sided colon cancer (RCC) have received increasing attention due to the clinicopathological variation between them. However, some of these differences have remained unclear and conflicting results have been reported.

**Methods:**

From The Cancer Genome Atlas (TCGA), we obtained RNA sequencing data and gene mutation data on 323 and 283 colon cancer patients, respectively. Differential analysis was firstly done on gene expression data and mutation data between LCC and RCC, separately. Machine learning (ML) methods were then used to select key genes or mutations as features to construct models to classify LCC and RCC patients. Finally, we conducted correlation analysis to identify the correlations between differentially expressed genes (DEGs) and mutations using logistic regression (LR) models.

**Results:**

We found distinct gene mutation and expression patterns between LCC and RCC patients and further selected the 30 most important mutations and 17 most important gene expression features using ML methods. The classification models created using these features classified LCC and RCC patients with high accuracy (areas under the curve (AUC) of 0.8 and 0.96 for mutation and gene expression data, respectively). The expression of PRAC1 and BRAF V600E mutation (rs113488022) were the most important feature for each model. Correlations of mutations and gene expression data were also identified using LR models. Among them, rs113488022 was found to have significance relevance to the expression of four genes, and thus should be focused on in further study.

**Conclusions:**

On the basis of ML methods, we found some key molecular differences between LCC and RCC, which could differentiate these two groups of patients with high accuracy. These differences might be key factors behind the variation in clinical features between LCC and RCC and thus help to improve treatment, such as determining the appropriate therapy for patients.

## Background

Colorectal cancer (CRC) is a common and lethal disease. Although its mortality has been declining since 1990, its mortality rate is currently approximately 1.7–1.9% [1]. CRC remains the third most common cancer according to the World Health Organization. This disease can be characterized based on the embryological origin [2]. Right-sided colon cancer (RCC) originates from the midgut, including the cecum, ascending colon, and hepatic flexure. In contrast, left-sided colon cancer (LCC) originates from the hindgut, including the splenic flexure, descending colon, and sigmoid colon [3]. Over the past few years, the differences between LCC and RCC have received increasing attention due to their different prognoses, outcomes, and clinical responses to chemotherapy. In many publications, it has been reported that there are significant differences regarding the mutations, epidemiology, survival, pathology, and clinical presentation between RCC and LCC [4, 5]. Compared with LCC, RCC was reported to occur more in older patients and females, having a poorer prognosis [6]. RCC tumors were also reported to be poorly differentiated, and to be larger and have more advanced stages [4, 7, 8]. However, some conflicting results concerning the differences between RCC and LCC were reported, and it remains a topic of considerable debate whether tumor location itself has a significant impact on prognosis [7]. Furthermore, the differences in molecular features between LCC and RCC remain unclear [9]. Studies have found that BRAF was preferentially mutated in RCC, while epidermal growth factor receptor (EGFR) was generally amplified in LCC [10]. Several studies have also reported that mutations and protein expression of p53 differed significantly between LCC and RCC [11–13]. However, another study showed that p53 protein expression had no significant difference between LCC and RCC [14].

In light of this background, there is a need to comprehensively survey the differences of gene mutations and expression levels between LCC and RCC. Knowledge of the differences at the molecular level would help us to obtain an in-depth understanding of LCC and RCC and further improve their diagnostic and treatment strategies in clinical practice. The rapid development of high-throughput sequencing technologies has provided us with opportunities to characterize the diverse array of genomic changes found within each cancer type. Projects like The Cancer Genome Atlas (TCGA) have compiled mutation, gene expression, methylation, and copy number data across cancer types [15].

As demonstrated by many researchers, machine learning (ML) is becoming increasingly important in cancer prognosis and prediction. In this context, we here established a study to use ML methods to explore gene mutation and expression data from TCGA to infer the molecular differences between LCC and RCC.

## Methods

### Data collection

We initially downloaded gene mutation data of 283 colon cancer patients (from tumor tissue) from TCGA data portal. Next, we added clinical information of each patient to the mutation file using the unique patient ID. Among these patients, 112 had LCC and 171 had RCC. All level 3 mRNA expression (FPKM) and raw count data of 323 colon cancer patients (from tumor tissue) were also obtained from TCGA, of whom 189 had RCC and 134 had LCC. The descending colon, sigmoid colon, and splenic flexure of colon were classified as LCC and the ascending colon, cecum, and hepatic flexure of colon were classified as RCC [3].

### Differential analysis and annotation

Analysis of the differential expression of genes was implemented using the R package DEseq2 using raw count data [16]. Genes with adjusted *P*-values of less than 0.01 and absolute values of log2 fold change (log_2_FC) above 1 were considered to be differentially expressed genes (DEGs). Fisher’s exact test was used to calculate the significance of differences in the frequency of each mutation between LCC and RCC samples. Annotation of the mutations was conducted by ANNOVAR [17]. The pathway annotation of DEGs was performed using the R package clusterProfiler with adjusted *P*-values less than 0.05 [18]. Mann-Whitney test was used to compare the difference of mutation number between LCC and RCC. To compare the mean mutation number of each sample among LCC and RCC groups, we divided the total number of mutations in each group by sample size.

### Machine learning methods

Extreme Gradient Boosting (XGBoost) is a boosted tree method that is often used for supervised learning problems [19]. It has excellent scalability and performance and has become an outstanding machine learning method in many fields of study. In this study, we attempted to use it to classify LCC and RCC patients based on mutation data and gene expression data (FPKM). The mutations existing in at least two samples and DEGs were separately used as raw features for XGBoost to do the following feature selection. To find the most appropriate feature number, for each iteration, we fed XGBoost with different number of features and evaluated its performance using the mean area under the curve (AUC) score of 100 times 10-fold cross-validation. The selected features were further used to construct the final classifier model. Owing to the relatively small sample sizes, we controlled the complexity of the models to avoid overfitting; an L2 regularization term was applied and the maximum depth of each tree was set to 3. Other hyper-parameters in the XGBoost models were assigned the default settings. For each type of data, 65% of samples were randomly selected as a training dataset, and the remaining 35% of samples were selected as a testing dataset using a stratified sampling method. AUC was used to evaluate the models. All of the functions were accomplished using the Python package Scikit-learn (sklearn). The significance of AUC was estimated by a permutation test using the R package sigr.

### Network construction

We used STRING to identify the correlations between all DEGs and genes with selected mutations by XGBoost, as shown in Cytoscape [20, 21]. Owing to the fact that mutation features were discrete variables and gene expression data were continuous variables, we analyzed the correlations of mutations with DEGs using logistic regression (LR), the type of colon cancer (LCC or RCC) was used as a confounder. If the false discovery rate (FDR) of the coefficient for each gene (x) to a mutation (y) was below 0.05, it was considered that a significant correlation existed between the gene and mutation. The idea behind using LR to calculate correlations between the two types of variable is that, if there is a relationship between continuous and discrete variables, an accurate predictor of the discrete variable would be constructed using the continuous variable. If the coefficient of the variable in the model is significant, we can conclude that the two variables have a relationship and are indeed correlated. The LR models were constructed using the R function glm.

## Results

### The 30 most important mutations classifying LCC and RCC

Among the 283 samples, there were 169,298 mutations in total. The mean number of mutations in each sample of RCC group was 2.86 times that in LCC (*P*-value< 0.001). We initially used Fisher’s exact test to calculate the significance of the difference between LCC and RCC samples in the frequency each mutation. The results are shown in Fig. S1. Among the mutations, the most significant ones were rs113488022 (BRAF, V600E, *P*-value, 4.72e-15) and rs112445441 (KRAS, *P*-value, 8.74e-11). The rs113488022 mutation was only found in 2 (1.8%) LCC patients, but in 35 (20.5%) RCC patients. The BRAF V600E mutation was reportedly found in 8–10% of colorectal tumors and was associated with a more aggressive tumor phenotype, lymph node metastasis, and high microsatellite instability (MSI) [20]. It was also found to be associated with less benefit from treatment [22]. In our study, the rs112445441 mutation in the KRAS gene was present in 8 LCC (7.1%) patients and 26 RCC (15.2%) patients.

**Fig. 1:**
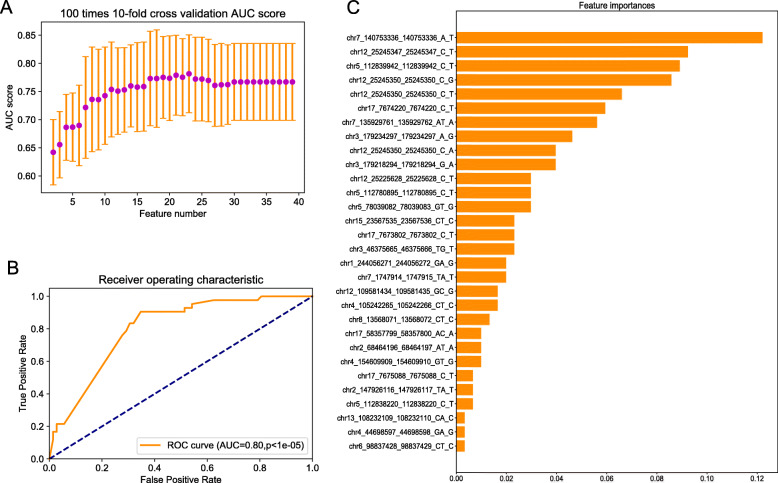
Feature selection and classification model based on mutations between LCC and RCC. **a** AUC scores of 100 times 10-fold cross-validation using different feature numbers. **b** The ROC curve of the classification model on the test dataset. **c** The importance score of the 30 mutation features

**Fig. 2:**
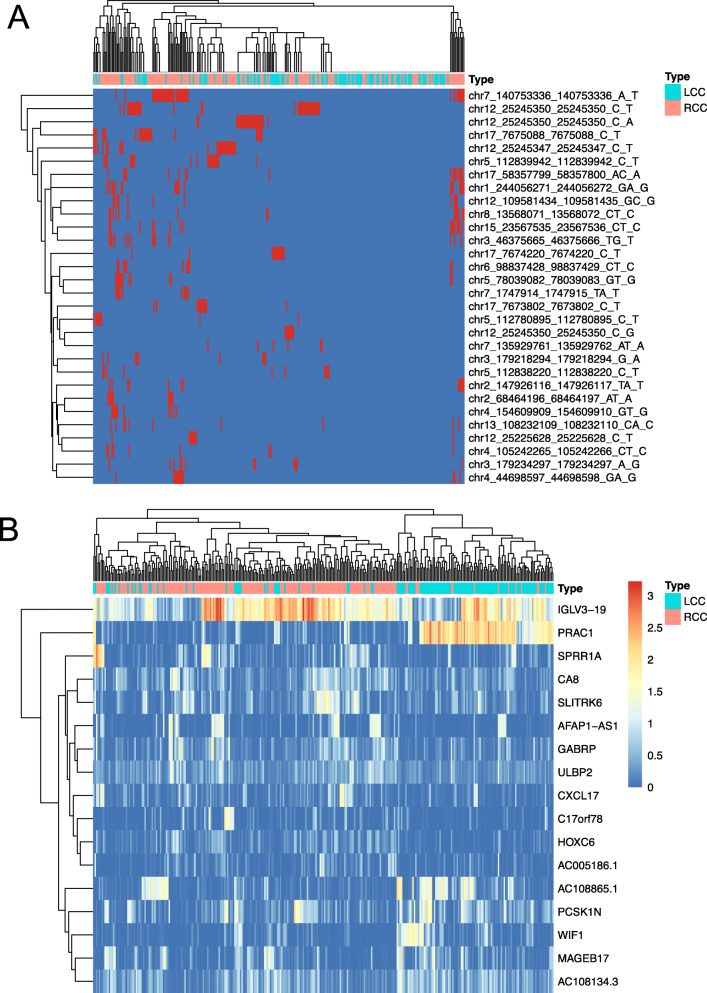
Heatmaps of the selected mutations and gene expression data. **a** Information of 30 mutations in LCC and RCC samples. Red represents an mutation being present in the sample, while blue represents no corresponding mutation in the sample. **b** Gene expression of 17 DEGs in LCC and RCC samples. Color represents log_10_(FPKM+ 1)

To select the most appropriate feature number to construct the model that could classify LCC and RCC patients, the AUC scores of 100 times 10-fold cross-validation for models with different feature numbers were obtained, as shown in Fig. 1a. Finally, we chose 30 features to construct the final model; its AUC score in the test dataset was 0.8 (*p* < 1e-05). Receiver Operating Characteristic (ROC) curves and the importance score of the 30 features are shown in Fig. 1b and c. The BRAF V600E mutation was the most important feature for the classifier model. Detailed information and the heatmap of the 30 mutation features are presented in Table 1, Supplementary File 1 and Fig. 2a.

**Table 1:** Information on 30 mutation features

Mutation^a^	avsnp150^b^	Gene.refGene^c^	weights^d^	Sample Number in LCC(%)^e^	Sample Number in RCC_(%)^f^	*P* value^g^
chr7_140753336_140753336_A_T	rs113488022	BRAF	0.12	2 (1.8)	35 (20.5)	4.72E-15
chr12_25245347_25245347_C_T	rs112445441	KRAS	0.09	5 (4.5)	20 (11.7)	4.07E-08
chr5_112839942_112839942_C_T	rs121913332	APC	0.09	1 (0.9)	18 (10.5)	2.93E-07
chr12_25245350_25245350_C_G	rs121913529	KRAS	0.09	1 (0.9)	6 (3.5)	0.02
chr12_25245350_25245350_C_T	rs121913529	KRAS	0.07	8 (7.1)	26 (15.2)	8.74E-11
chr17_7674220_7674220_C_T	rs11540652	TP53	0.06	10 (8.9)	2 (1.2)	0.56
chr7_135929761_135929762_AT_A	.	LUZP6;MTPN	0.06	4 (3.6)	2 (1.2)	0.56
chr3_179234297_179234297_A_G	rs121913279	PIK3CA	0.05	5 (4.5)	7 (4.1)	0.01
chr12_25245350_25245350_C_A	rs121913529	KRAS	0.04	11 (9.8)	13 (7.6)	3.46E-05
chr3_179218294_179218294_G_A	rs121913273	PIK3CA	0.04	4 (3.6)	5 (2.9)	0.04
chr12_25225628_25225628_C_T	rs121913527	KRAS	0.03	2 (1.8)	6 (3.5)	0.02
chr5_112780895_112780895_C_T	rs587781392	APC	0.03	4 (3.6)	4 (2.3)	0.08
chr5_78039082_78039083_GT_G	.	AP3B1	0.03	2 (1.8)	10 (5.8)	5.37E-4
chr15_23567535_23567536_CT_C	.	MKRN3	0.02	1 (0.9)	15 (8.8)	5.29E-06
chr17_7673802_7673802_C_T	rs28934576	TP53	0.02	5 (4.5)	6 (3.5)	0.02
chr3_46375665_46375666_TG_T	rs939905165	LOC102724297	0.02	0 (0)	15 (8.8)	5.29E-06
chr1_244056271_244056272_GA_G	rs972665297	ZBTB18	0.02	2 (1.8)	17 (9.9)	7.77E-07
chr7_1747914_1747915_TA_T	.	ELFN1	0.02	2 (1.8)	8 (4.7)	3.08E-3
chr12_109581434_109581435_GC_G	.	MVK	0.02	0 (0)	11 (6.4)	2.16E-4
chr4_105242265_105242266_CT_C	.	TET2-AS1	0.02	1 (0.9)	8 (4.7)	3.08E-3
chr8_13568071_13568072_CT_C	rs1014242184	C8orf48	0.01	1 (0.9)	14 (8.2)	1.36E-05
chr17_58357799_58357800_AC_A	rs781215815	RNF43	0.01	0 (0)	17 (9.9)	7.77E-07
chr2_68464196_68464197_AT_A	.	FBXO48	0.01	0 (0)	9 (5.3)	1.29E-3
chr4_154609909_154609910_GT_G	.	FGG	0.01	0 (0)	10 (5.8)	5.32E-4
chr17_7675088_7675088_C_T	rs28934578	TP53	0.01	10 (8.9)	13 (7.6)	3.46E-05
chr2_147926116_147926117_TA_T	rs764719749	ACVR2A	0.01	1 (0.9)	11 (6.4)	2.16E-4
chr5_112838220_112838220_C_T	rs121913333	APC	0.01	3 (2.7)	7 (4.1)	7.22E-3
chr13_108232109_108232110_CA_C	rs977361714	ABHD13	0.003	1 (0.9)	9 (5.3)	1.29E-3
chr4_44698597_44698598_GA_G	.	GUF1	0.003	1 (0.9)	11 (6.4)	2.16E-4
chr6_98837428_98837429_CT_C	rs898072886	POU3F2	0.003	1 (0.9)	10 (5.8)	5.32E-4

^a^Position of variants. For example, chr7_140753336_140753336_A_T represents base A being replaced by T at position 140,753,336 of chromosome 7

^b^The annotation of variants with dbSNP identifiers by ANNOVAR

^c^The annotated genes of the variants by ANNOVAR

^d^The weights (importance) of the mutation features for the classification model

^e^The number of samples (percent of samples) with the variants among LCC samples

^f^The number of samples (percent of samples) with the variants among RCC samples

^g^The *P*-value from Fisher’s exact test for each variant

### The 17 most important DEGs classifying LCC and RCC

**Fig. 3:**
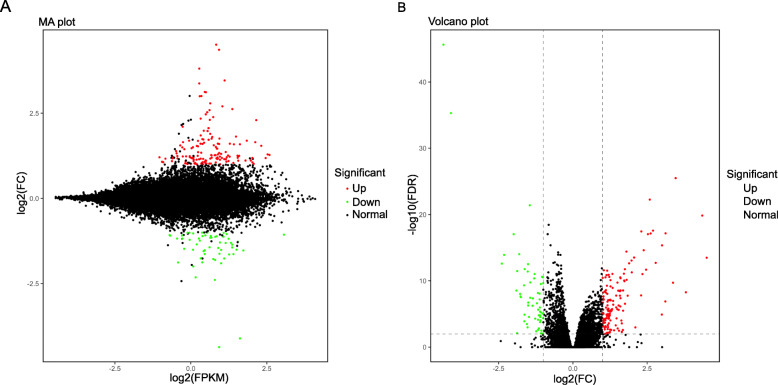
Volcano plot and MA plot for DEGs. Red dots represent upregulated genes in RCC compared with the level in LCC, while green dots represent downregulated genes

**Fig. 4:**
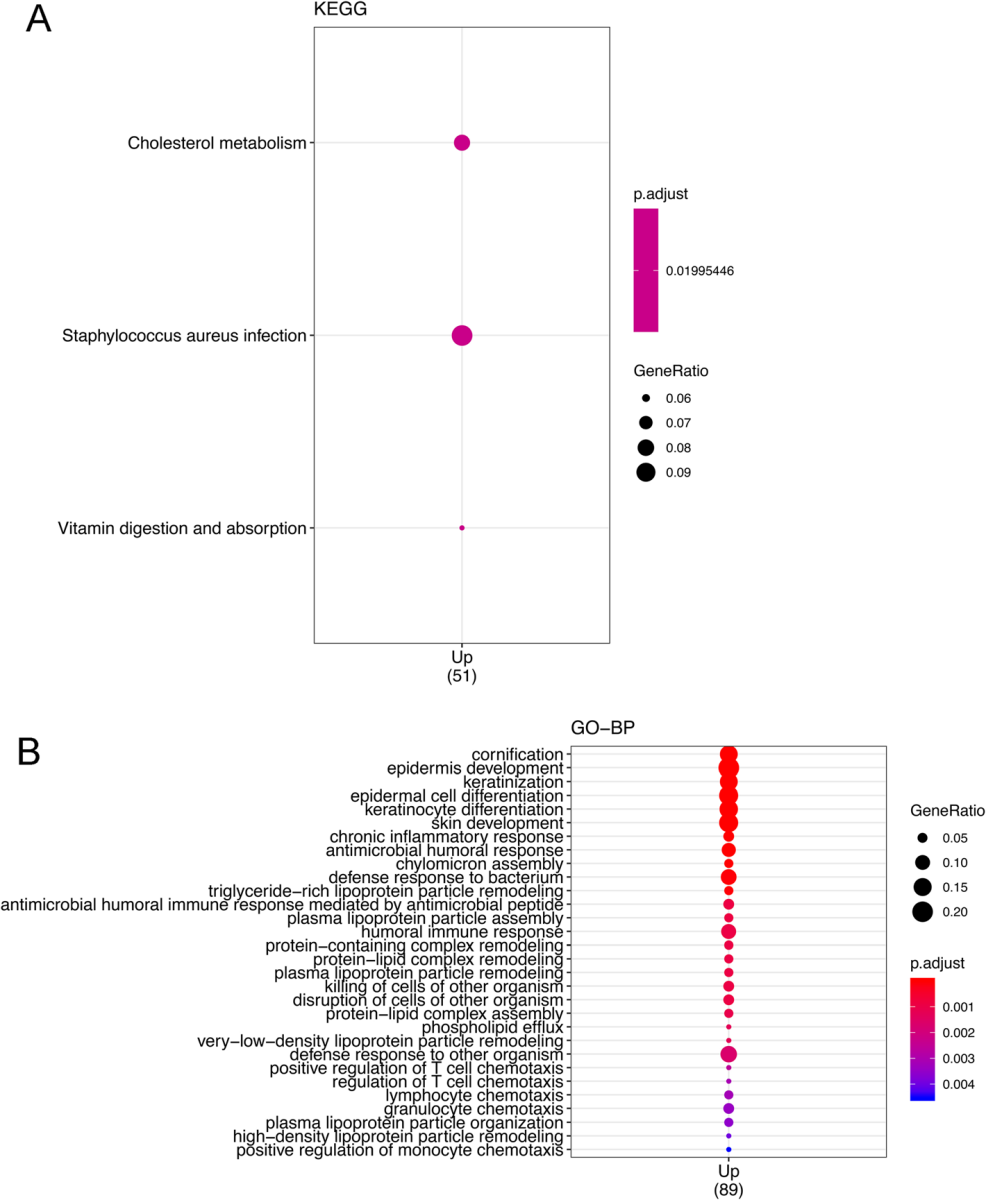
Annotation results of DEGs in KEGG (**a**) and GO analyses (**b**). Only genes that were upregulated in RCC compared with the level in LCC were enriched in KEGG pathways and GO analyses (adjusted *P*-value< 0.05)

Overall, 144 genes were upregulated and 60 were downregulated in RCC compared with the levels in LCC. A volcano plot and MA plot of DEGs are shown in Fig. 3. The genes with a higher expression level in RCC were particularly associated with the vitamin digestion and absorption pathway, cholesterol metabolism pathway, and *Staphylococcus aureus* infection pathway (Fig. 4a). The Gene Ontology (GO) annotation results for the main category of biological process (BP) are shown in Fig. 4b. Among these results, some immunity-related processes such as positive regulation of T-cell chemotaxis (GO:0010820), which could increase the rate, frequency, or extent of T-cell chemotaxis; lymphocyte chemotaxis (GO:0048247), which could direct the movement of a lymphocyte in response to an external stimulus; and granulocyte chemotaxis (GO:0071621), which could induce the movement of a granulocyte in response to an external stimulus, were particularly associated with the upregulated DEGs.

**Fig. 5:**
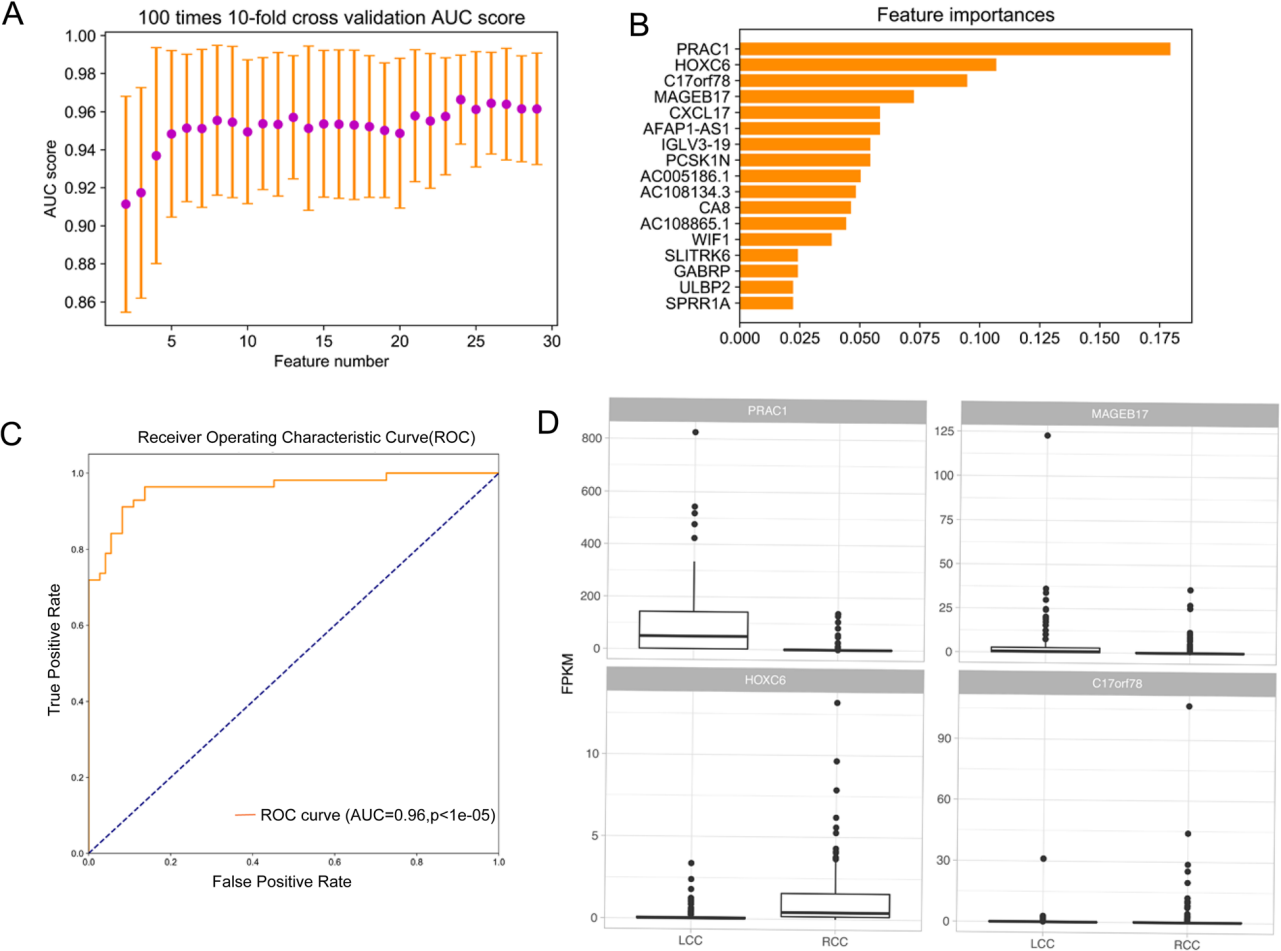
Feature selection and classification model based on DEGs between LCC and RCC. **a** AUC scores of 100 times 10-fold cross-validation using different feature numbers. **b** The importance score of the 30 mutation features. **c** The ROC curve of the classification model on the test set. **d** Boxplot of the top four genes with the highest importance score in LCC and RCC

On the basis of these DEGs, we further selected 17 features to construct a model that could accurately classify LCC and RCC samples. Figure 5a shows the AUC scores of 100 times 10-fold cross-validation, with the feature number varying from 2 to 30. For the final model, the AUC score in the test dataset (35%) was 0.96 (Fig. 5c, *p* < 1e-5). Among the 17 features, PRCA1 obtained the highest score for the model (Fig. 5b); PRCA1 is a novel small nuclear protein that is specifically expressed in the human prostate and colon [23]. The relative expression value (Fragments Per Kilobase Million, FPKM) of the top 4 genes with the highest scores from the classifier model among LCC and RCC groups is shown in Fig. 5d. The FPKM of all 17 genes in the LCC and RCC groups are shown in Fig. S2. A heatmap of the expression of the 17 genes in LCC and RCC is shown in Fig. 2b.

### Analysis of the correlations of DEGs with mutations

**Fig. 6:**
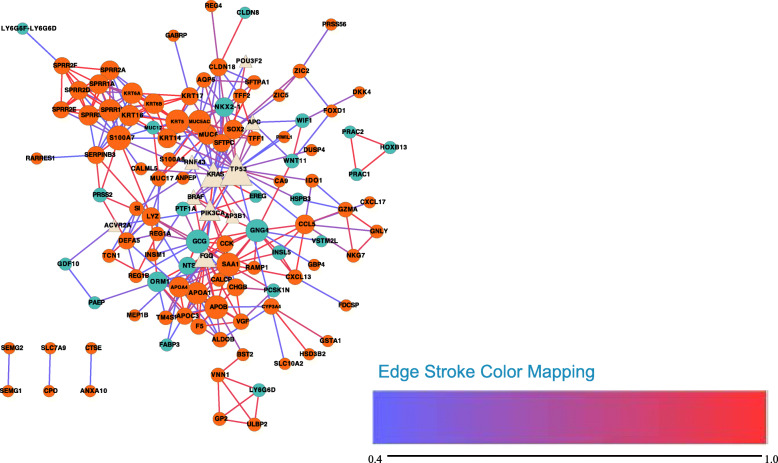
Network of all of the DEGs and genes with the selected 30 mutations (Produced by Cytoscape Version 3.7.1). Circle nodes represent DEGs, while triangles represent mutated genes. Nodes with a light yellow color represent genes with mutations, dark turquoise represents downregulated DEGs, while dark orange represents upregulated DEGs. The line color represents the score of the connection between two nodes, ranging from 0.4 to 0.99. Node size represents the degree of the node: the larger the node size, the higher the degree of the node

A network of the correlations of all DEGs with mutant genes (genes containing the 17 selected mutations) was constructed using STRING [20] (Fig. 6). TP53 and KRAS are hub genes that were found to be connected to many DEGs such as WIF1 and KRT17.

**Fig. 7:**
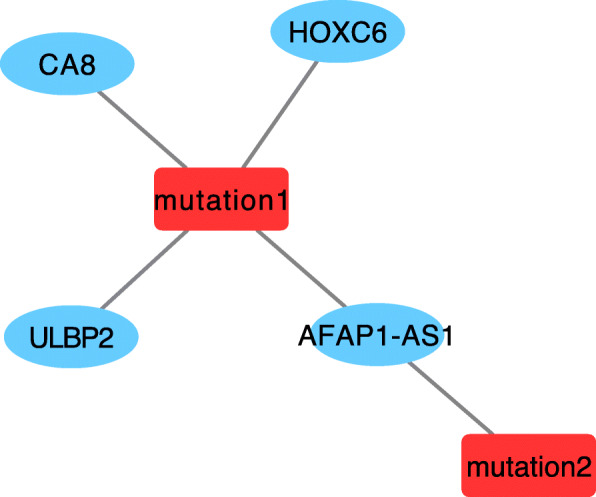
The correlation network of 30 mutations and 17 DEGs calculated by logistic regression model (FDR < 0.05, Produced by Cytoscape Version 3.7.1). Nodes with red color represent mutations, mutation1 represents rs113488022 (BRAF, V600E mutation), mutation2 represents mutation in the 3′-UTR of ELFN1(chr7_1747914_1747915_TA_T- represents the chromosome, the position, and the mutated base). Nodes with a blue color represent DEGs

The correlations of 30 mutations with 17 DEGs selected by ML were determined by logistic regression (LR) and visualized using Cytoscape [21] (Fig. 7). Five relationships were found (two mutations, four DEGs, FDR < 0.05). Among them, the BRAF V600E mutation was correlated with four DEGs (ULBP2, CA8, HOXC6, AFAP1-AS1). The coefficients and FDR values of the correlations for BRAF V600E mutation to the four genes are listed in Table 2.

**Table 2:** The correlations of rs113488022 with DEGs

Mutation	Gene	Coefficient^a^	FDR^a^
rs113488022	ULBP2	0.31	0.01
	CA8	0.18	0.04
	HOXC6	0.68	0.002
	AFAP1-AS1	0.13	0.001

^a^The coefficients and adjusted *P*-values (FDR) of the correlations of rs113488022 with genes from logistic regression model

## Discussion

It has been hypothesized that there are significant differences between RCC and LCC in terms of the molecular features, which might be the cause of clinicopathological differences [24]. However, the differences of molecular features between RCC and LCC patients have remained unclear. Using ~ 300 LCC and RCC samples from TCGA, we attempted to unearth more valuable information on the differences between LCC and RCC by applying ML methods. It has been reported that RCC has a higher incidence of KRAS mutation than LCC (57.3% vs. 40.4%; *P*-value < 0.0001) [25], and a higher frequency of BRAF mutation (18.4–22.4% vs. 1.3–7.8%) [26]. However, other studies found no significant differences in BRAF and KRAS mutation rates [27]. In our study, RCC was also found to have higher incidences of KRAS mutation (49.7% vs. 33.0%, *P*-value = 0.007) and BRAF mutation (23.4% vs. 3.6%, *P*-value = 2.8e-6) than LCC. However, no significant difference was found in the expression of BRAF (FDR = 1, log_2_FC = 0.1) and KRAS (FDR = 0.92, log_2_FC = − 0.04) in our study, which implies that the mutations may have no impact on the transcription of KRAS and BRAF. The top four genes with the highest mutation rates in LCC were APC (84.8%), TP53 (68.8%), TTN (54.5%), and KRAS (33%). In RCC, the top four again included APC as the most common (63.2%), followed by TTN (63.2%), KRAS (49.7%), and TP53 (49.7%).

Using ML methods, we selected 30 mutations to build an XGBoost classifier with AUC of 0.80 in the test dataset. The feature with the highest score in the model was rs113488022 in BRAF. The top seven mutations scored by XGBoost were in the BRAF, KRAS, APC, and TP53 genes. APC and TP53 are tumor suppressor genes, while KRAS and BRAF are oncogenes. The differences in the frequencies of these mutations may be the reason for the clinicopathological differences between the two types of colon cancer.

The genes that were upregulated in RCC compared with the levels in LCC were particularly associated with some immunity-related processes. Using DEGs, we constructed a model with AUC of 0.96 in the test set using only 17 features, implying large differences between LCC and RCC at the level of gene expression. Among these features, small nuclear protein PRAC1 (FDR < 0.001, log_2_FC = − 4.1) was the most important, which was highly expressed in LCC. The higher expression of PRAC1 in LCC than RCC was also identified in other studies [5]. However, the function of PRAC1 in colon cancers remains elusive. Mutations in this gene have been found to be associated with a predisposition to prostate cancer and it is a candidate for the hereditary prostate cancer 1 (HPC1) allele. The second most important feature in the model, HOXC6 (FDR < 0.001, log_2_FC = 1.03), was highly expressed in RCC; it belongs to the homeoprotein family of transcription factors, members of which play important roles in morphogenesis and cellular differentiation during embryonic development [28]. The higher expression of HOXC6 in RCC than LCC was also described in another study [5]. Furthermore, the overexpression of HOXC6 has been detected in several human carcinomas, including breast, gastrointestinal, and lung cancers, as well as leukemia [29]. High expression levels of HOXC6 have also been found to be associated with lymph node metastasis [30]. The differential expression of PRAC1 and HOXC6 and other genes may be the reason for the different characteristics between LCC and RCC, which warrants more attention in further study.

In the correlation network, it was shown that some of the mutant genes, such as TP53 and KRAS, were the hub nodes. The LR analysis also showed a close relationship between BRAF V600E mutation and the expression of four genes such as HOXC6 and CA8. These findings suggest that the differences of gene mutations and expression, and the associations between them may be the key reasons for the differences in clinical features between LCC and RCC.

## Conclusions

In this study, we used ML methods to clarify some of the key molecular differences between LCC and RCC. Two classification models were constructed using the selected 17 DEGs and 30 mutations separately with good performance in the prediction of the two types of colon cancers. The expression of PRCA1 and the BRAF V600E mutation were the most important features for the two classifier models. Furthermore, BRAF V600E mutation was found to correlate with four genes among the 17 DEGs which should be paid more attention in further studies about colon cancer. Overall, the classifier models and the identified different mutations and genes in LCC and RCC might help us to obtain an in-depth understanding and further improve the diagnostic and therapy for patients.

## Supplementary information

**Additional file 1.**

**Additional file 2: Figure S1.** Comparison of mutation landscape between LCC and RCC. Each point represents a mutation, the x-axis represents the chromosomes, and the y-axis represents the negative of the base 10 logarithm of the *P*-values.

**Additional file 3: Figure S2.** The relative expression value (FPKM) of 17 DEGs in LCC and RCC.

## Data Availability

The analyzed datasets generated during the study are available from the corresponding author on reasonable request.

## Abbreviations

LCC: Left-sided colon cancer
RCC: Right-sided colon cancer
ML: Machine learning
LR: Logistic regression
TCGA: The Cancer Genome Atlas
EGFR: Epidermal growth factor receptor
XGBoost: Extreme Gradient Boosting
AUC: Area under the curve
sklearn: Scikit-learn
log2FC: log2 fold change
DEG: Differentially expressed gene
MSI: Microsatellite instability
ROC: Receiver Operating Characteristic
GO: Gene Ontology
BP: Biological process
FDR: False discovery rate
