# Supplementary figures and images for: Discovering the molecular differences between right- and left-sided colon cancer using machine learning methods
Source: BMC Cancer. 2020 Oct 19;20:1012. doi: 10.1186/s12885-020-07507-8 (PMC7574488; doi:10.1186/s12885-020-07507-8)

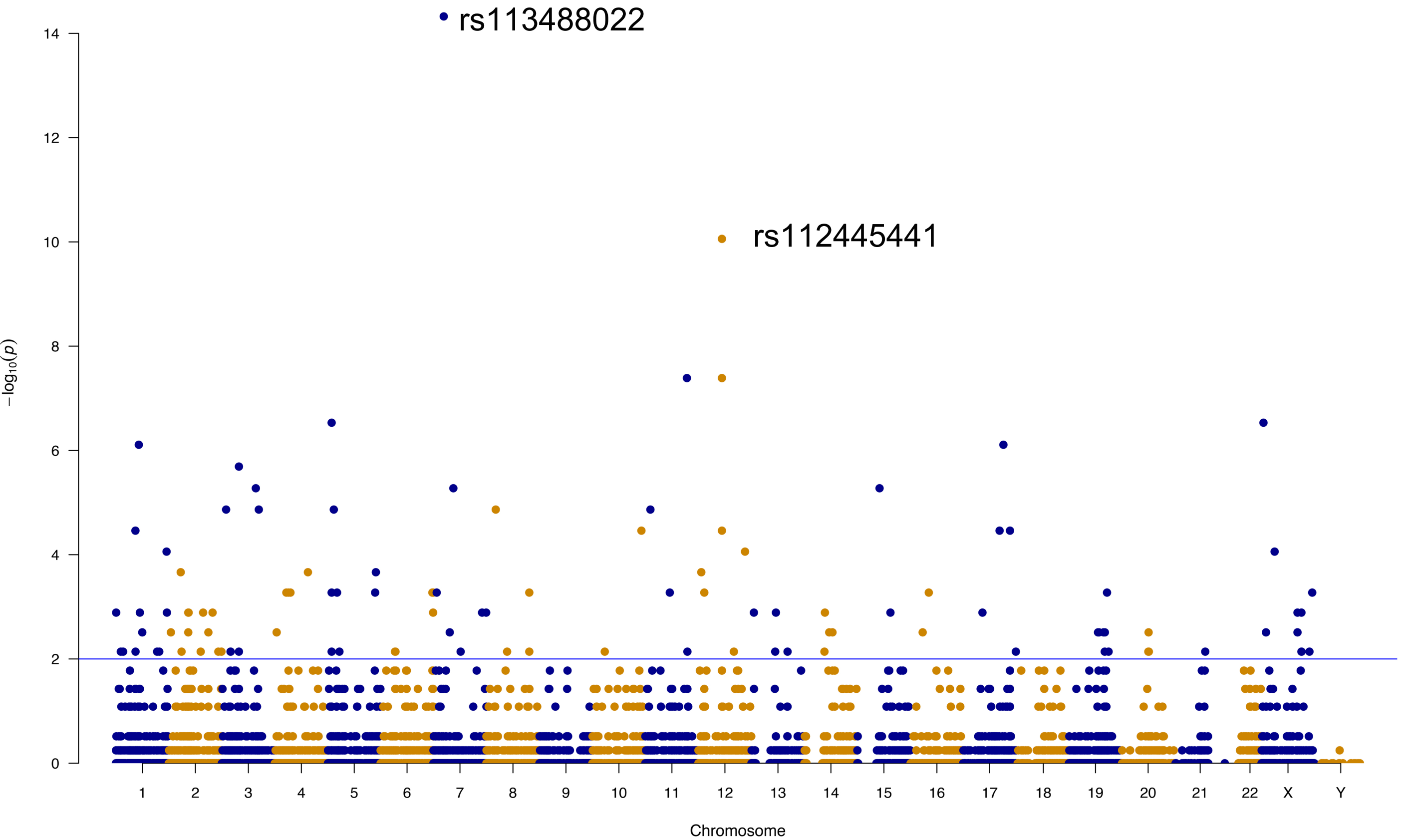

Supplement: Supplementary file 2 — Additional file 2: Figure S1. Comparison of mutation landscape between LCC and RCC. Each point represents a mutation, the x-axis represents the chromosomes, and the y-axis represents the negative of the base 10 logarithm of the P-values. [file 12885_2020_7507_MOESM2_ESM.pdf]

FPKM

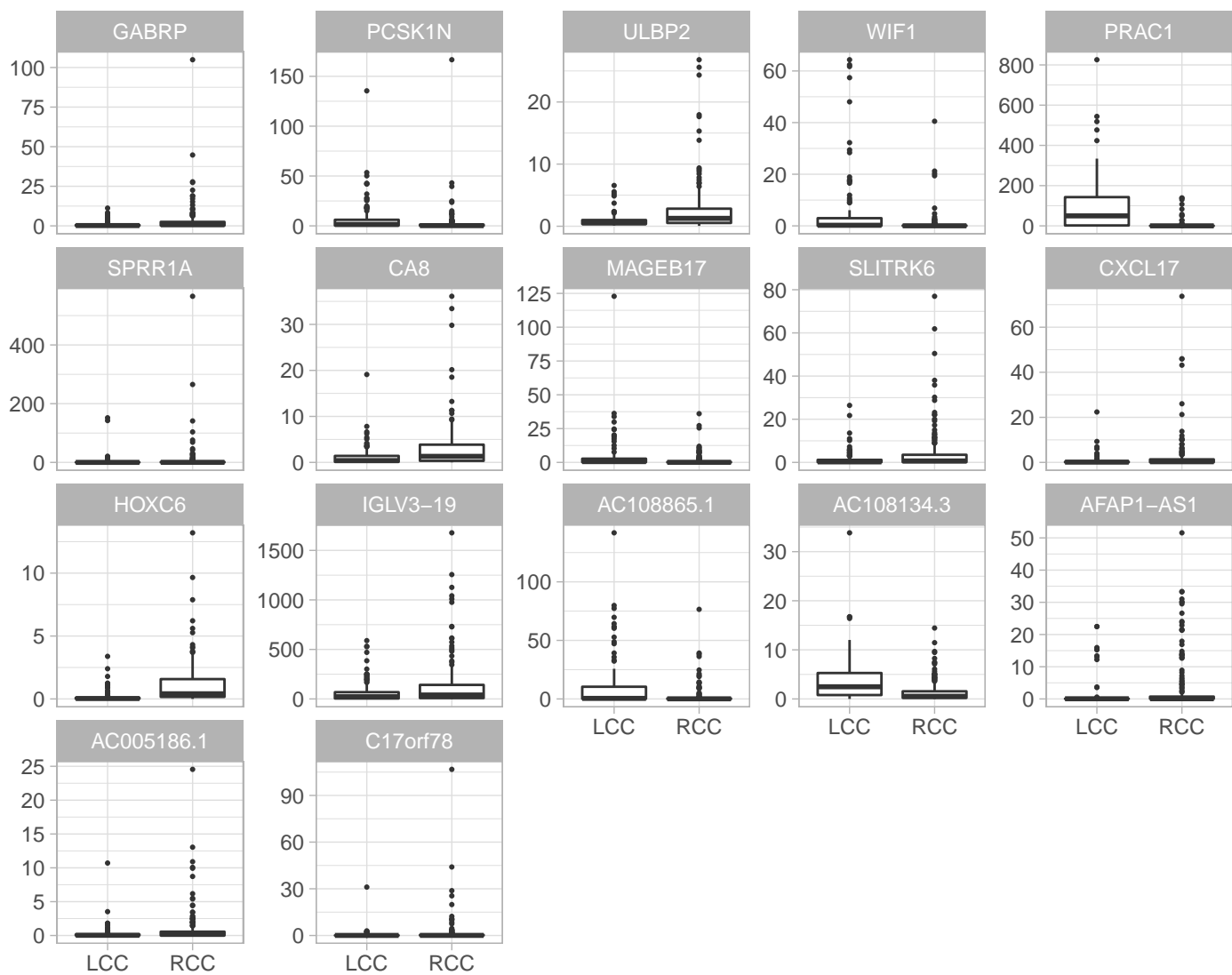

Supplement: Supplementary file 3 — Additional file 3: Figure S2. The relative expression value (FPKM) of 17 DEGs in LCC and RCC. [file 12885_2020_7507_MOESM3_ESM.pdf]
